# Obesity Among Pregnant Women in Saudi Arabia: A Retrospective Single-Center Medical Record Review

**DOI:** 10.7759/cureus.13454

**Published:** 2021-02-20

**Authors:** Anas M Fallatah, AlRayan AlNoury, Enas M Fallatah, Khalid M Nassibi, Hussam Babatin, Omar A Alghamdi, Badeyah Y Tarabaih, Hassan S Abduljabbar

**Affiliations:** 1 Medicine, College of Medicine, King Abdulaziz University, Jeddah, SAU; 2 Obstetrics and Gynecology, East Jeddah Hospital, Jeddah, SAU; 3 Medicine, College of Medicine, King Saud Bin Abdulaziz University for Health Sciences, Jeddah, SAU; 4 Medicine, College of Medicine, Ibn Sina National College, Jeddah, SAU; 5 Obstetrics and Gynecology, King Abdulaziz University, Jeddah, SAU

**Keywords:** prevalence, obesity, obstetric, pregnancy outcomes, jeddah, saudi arabia

## Abstract

Background

Obesity in Saudi Arabia is on the rise, especially among females who are more likely to suffer from obesity in the reproductive age group than males in the adult age group. Biologically, pregnancy can increase women’s weight and put them at greater risk for adverse obstetric outcomes.

Objectives

To find the prevalence of obesity among pregnant women and their obstetric outcomes.

Methods

This retrospective study was conducted on pregnant women who delivered between January 2013 and May 2018 at the obstetrics clinic of King Abdulaziz University Hospital (KAUH), Jeddah, Saudi Arabia. A datasheet was formed to collect data from the medical records of these pregnant women. The analysis was done using the Statistical Package for Social Sciences (SPSS), version 26 (IBM SPSS Statistics, Armonk, NY). A p-value of < 0.05 was used to calculate statistical significance.

Results

A total of 9,095 pregnant women delivered during that period. Of those women, a total of 2,235 were found to be obese, and 1,842 were included in the study. A total of 1,130 women were categorized under Class I obesity, 458 were categorized under Class II obesity, and 254 were categorized under Class III obesity. The majority of the sample were Saudis (72.3%) and young adults (90.8%) with 1,672 cases. The average age was 31.7 (standard deviation (SD): 5.9). Twenty percent of the sample had preterm newborns, while the majority (62.4%) ended up with cesarean delivery. Advanced age, multipara, and cesarean delivery were statistically significant with adverse pregnancy outcomes (p < 0.05).

Conclusion

As demonstrated in this study, obesity among females in Saudi Arabia has increased over the past decade. Hence, this puts them at higher risk of developing adverse pregnancy outcomes, as pregnancy physiologically results in additional weight gain. Proper antenatal counseling, health education, and a comprehensive plan prior to conception are highly recommended.

## Introduction

Obesity is a state of generalized adiposity, wherein an abnormal increase in the fat content of the body impacts negatively on general health [1]. Several anthropometric measurements are employed to assess obesity, such as waist circumference, waist-to-hip ratio, and body mass index (BMI), with comparable clinical utility. However, the BMI, which is obtained by dividing weight in kilograms by squared height in meters, is considered the most practical anthropometric measure of obesity. Further, BMI cut-off points are utilized in categorizing weight to four groups: underweight (less than 18.5 kg/m^2^), normal weight (18.5 - 24.9 kg/m^2^), overweight (25 - 29.9 kg/m^2^), and obesity (greater than or equal to 30 kg/m^2^) [2]. Moreover, obesity is subcategorized into three classes: Class I (BMI: 30 - 34.9), Class II (BMI: 35 - 39.9), and Class III (BMI ≥ 40).

In recent years, obesity has reached the levels of an epidemic in the middle-income, as well as low-income, countries [2]. Data released from 16 countries in the Eastern Mediterranean region have shown high numbers of obese adults aged 18 years and beyond, especially in Egypt, Bahrain, Jordan, Kuwait, Saudi Arabia (SA), and the United Arab Emirates [2]. Locally, in our community, obesity is seen at a higher level than other local communities in the Gulf region, reaching 36% of its general population [3]. Females had the highest proportion in the aforementioned study. Subsequently, obesity among females in the Western region in SA was 22%, according to a cross-sectional study [4]. Diseases that can result in significant morbidities, such as hypertension, heart diseases, diabetes mellitus type 2, rheumatological diseases, biliary tract diseases, and specific forms of cancer (e.g., endometrial cancer) are highly associated with obesity [5].

Pregnant obese women are at risk of many pregnancy complications, such as gestational diabetes mellitus, pregnancy-induced hypertension, preeclampsia, induction of labor, preterm labor, preterm birth, increased rates of cesarean section (CS), postpartum hemorrhage, anemia, urinary tract infection, wound infection, and prolonged pregnancy [6-10]. Additionally, obesity in pregnancy is linked to obstetric risks, such as shoulder dystocia, fetal macrosomia, perinatal death, fetal birth defects, and admission to the neonatal intensive care unit [7, 11-12].

The results of a systematic review of 42 multinational studies (but predominantly from Asia) examining the relationship between maternal BMI and risk of adverse and health outcomes have shown obesity to be associated with a greater risk of harmful maternal outcomes [13]. In addition, two studies in SA were carried out to describe the local frequencies of obesity (7, 14). A study conducted in the Eastern province showed that 28.7% of their population were obese, while in Buraida, it was 30% [7, 14].

Despite existing studies and those presented on obesity in pregnancy, none are from the Western region of SA, which encouraged the development of our research. This study's primary aim is to determine the prevalence of obesity among pregnant women attending the Obstetrics Clinic at King Abdulaziz University Hospital (KAUH), Jeddah, SA. A secondary aim is to correlate obesity during pregnancy with the outcome of those pregnancies. Outcomes in question are increased rates of CS, preterm, and post-term deliveries.

## Materials and methods

This retrospective study was conducted among pregnant women attending the Obstetrics Clinics at KAUH from January 2013 to May 2018. A datasheet was established to help the data collectors obtain relevant information from the electronic medical file records. After acquiring the ethical approval from the Institutional Review Board and the Research Ethics Committee of KAUH, the electronic records were obtained from the hospital’s Medical Records Department.

From that period, a total of 9,095 pregnant women attended our hospital. A total of 2,235 were obese. Of these, 1,842 were included for further analysis. Candidates with missing files or with multiple gestations were excluded from this study. The anthropometric measures were taken at their initial obstetric clinic visit. Obesity is categorized into three subclasses using their BMI: Class I (BMI: 30 - 34.9), Class II (BMI: 35 - 39.9), and Class III ( BMI ≥ 40) [2]. Age was subdivided into two groups: young adults (15 - 39) and middle-aged adults (40 - 60). 

Obstetric variables that were addressed as adverse pregnancy outcomes were gestational age and mode of delivery. Gestational age was determined by the last menstruation period, or craniocaudal length calculated by ultrasound during the first trimester of pregnancy. Preterm, full-term, and post-term labeling was done according to the recent guidelines of the American College of Obstetrics and Gynecology (ACOG) in 2013 [15].

Data were initially revised and organized in Microsoft Excel for Mac, version 16 (Microsoft® Corp., Redmond, WA, USA). The IBM Statistical Package for Social Sciences (SPSS) for Macintosh operating system (macOS), version 23.0 (IBM SPSS Statistics for Macintosh, Armonk, NY, USA) was used for data analysis. Frequencies and percentages were calculated for categorical variables, and central tendency measures were calculated for the continuous variables. Further statistical modalities were used, such as the Chi-square test, independent t-test, one-way analysis of variance (ANOVA), and Pearson’s correlation. A p-value of less than 0.05 was considered significant.

## Results

This study revealed that out of 1,842 obese pregnant women, 1,331 (72.3%) were Saudis and 511 (27.7%) were non-Saudis. It also revealed that the mean age was 31.7 years (standard deviation (SD): 5.76). Also, the mean height was 156.2 cm (SD: 6.25), and the mean weight was 85.4 kg (SD: 12.95). The BMI at the first clinic visit of all obese pregnant women showed a mean of 34.9 (SD: 4.54). Also, the mean of gravidity and parity showed a value of 3.91 (SD: 2.19) and 2.4 (SD: 1.82), respectively. The mean gestational age was 38.1 weeks (SD: 2.66). Regarding age groups, the majority were young adults (90.8%), while 170 (9.2%) were middle-aged adults. Also, the obese Class I was the most prevalent with 1,130 cases. Moreover, preterm newborns were seen in 20% of the sample with 306 cases. Concerning the mode of delivery, most patients ended up with CS delivery with 1,150 cases vs 692 cases who had a spontaneous vaginal delivery (SVD).

Table 1 presents the sociodemographic characteristics of the participants among the obese classes. It revealed that obesity among Saudis was higher than non-Saudis, with a percentage of 72.3%, and most of them were in Class I with 806 (71.3%). Also, it showed that Class I had the majority of those who were multipara (more than two) with 449 (39.8%). The study also found a significant association between parity and obesity (P = 0.001), with a positive correlation (correlation coefficient (r) = 0.076). Moreover, the study found a positive correlation that was statistically significant between age and obesity, with (r = 0.86) and (P = 0.000), respectively. This study also showed that most young adults were in obese Class I (1,031, 91.2%) compared to middle-aged adults.

**Table 1:** Sociodemographic Characteristics of the Pregnant Women BMI: body mass index; n: sample size; SD: standard deviation

		Obese Class I	Obese Class II	Obese Class III	P-value
		(BMI: 30 - 34.9)	(BMI: 35 - 39.9)	(BMI ≥ 40.0)	
		N = 1,130	N = 458	N = 254	
Age	Mean (SD)	31.45 (5.76)	31.72 (5.83)	32.86 (5.49)	0.000
Height	Mean (SD)	156.32 (6.25)	156.01 (5.93)	156.27 (6.75)	-
Weight	Mean (SD)	78.67 (7.02)	89.93 (7.53)	107.36 (13.25)	-
BMI	Mean (SD)	32.16 (1.39)	36.92 (1.38)	43.9 (3.91)	-
Nationality	Saudi (n = 1331)	806 (71.3%)	336 (73.4%)	189 (74.4%)	0.508
	Non-Saudi (n = 511)	342 (28.7%)	122 (26.6%)	65 (25.6%)	
Age Groups	Young adults (n = 1,672)	1,031 (91.2%)	414 (90.4%)	227 (89.4%)	0.616
	Middle age adults (n = 170)	99 (8.8%)	44 (9.6%)	27 (10.6%)	
Parity (n = 1,035)	None	144 (12.7%)	60 (13.1%)	25 (9.8%)	0.396
	One	280 (24.8%)	96 (21.0%)	60 (23.6%)	
	Two	257 (22.7%)	100 (21.8%)	52 (20.5%)	
	More than two	449 (39.8%)	202 (44.1%)	117 (46.1%)	

A total of 1,150 (62.4%) out of 1,842 patients had CS; most of them were in Class I (n: 639, 56.5%). However, SVD was higher among the obese Class I (n: 491, 43.5%). Additionally, this study showed a significant association between the mode of delivery and obesity (P= 0.000) (Table 2). The majority of our sample had full-term neonates, and most of them were in Class 1. Preterm neonates were more prevalent in the obese Class I compared to other classes. Also, post-term neonates were seen to be higher in the obese Class I. A negative correlation was found between gestational age and obesity (r = 0.038).

**Table 2:** Obstetric Outcomes Among Our Sample CS: cesarean section; n: sample size; SVD; spontaneous vaginal delivery

		Obese Class I	Obese Class II	Obese Class III	Total	P-Value
		(BMI: 30 - 34.9)	(BMI: 35 - 39.9)	(BMI ≥ 40.0)		
		N = 1,130	N = 458	N = 254		
Mode of Delivery	SVD (n = 692)	491 (43.5%)	138 (30.1%)	63 (24.8%)	692	0.000
	CS (n = 1,150)	639 (56.5%)	320 (69.9%)	191 (75.2%)	1,150	
Gestational Age (n = 1,035)	Preterm (n = 306)	185 (16.4%)	78 (17.0%)	43 (16.9%)	306	0.598
	Full-term (n = 1,488)	920 (81.4%)	367 (80.1%)	201 (79.1%)	1,488	
	Post-term (n = 48)	25 (2.2%)	13 (2.8%)	10 (3.9%)	48	

## Discussion

Obesity has become one of the most crucial health issues worldwide. It is a well-known health crisis in most developed countries and a significant problem in developing countries. Globally, more than one-third of the world’s population are impacted by obesity [16]. Additionally, it contributes to major health issues, such as cardiovascular diseases, diabetes, and cancers. The economic burden of obesity is similarly substantial. Further, an “obesogenic environment” was introduced into Arabic-speaking countries as a result of the rapid regional modernization in the last decade. This shift facilitated new ways of living (in terms of transportation and the proliferation of Western-style fast food) which led to a sedentary lifestyle [17].

Globally, according to a recent study conducted in 2018, a total of 1.9 billion and 609 million adults were estimated to be overweight and obese in 2015, respectively, representing approximately 39% of the world’s population [18]. In terms of gender distribution, the prevalence of obesity was generally higher in women than in men in all age groups, with sex differences being maximal between 50 and 65 years old [19]. To illustrate, a recent survey was conducted, and it showed that 44% of the female population and 28% of the male population in SA were obese [20]. However, 71% of women and 66% of men were overweight.

As this study showed, a total of 2,235 (25%) were found to be obese, which is in keeping with the findings of a study conducted in Al-Hassa with a prevalence of 29% [7]. Also, the present study’s finding was consistent with that of a study conducted in Buriada, where 30% of their sample was found to be obese [21]. Internationally, a study conducted in Beijing, China showed that the prevalence of overweight and obesity in pregnant women was 9.61% [22]. In contrast, data from the Health Survey from England showed that the prevalence of obesity in women was 23.0% in 2005 and 23.9% in 2009 [23]. International comparisons of obesity are considered a challenge due to the heterogeneity in sampling and design; thus, direct, precise comparisons of local and global rates are limited [23]. 

According to our previous study, along with another study conducted in India, obese pregnant women are at higher risk of developing intrapartum and postpartum complications, such as gestational hypertension, gestational diabetes, and preeclampsia. Additionally, they were at higher risk of developing intrapartum complications, namely increased rates of cesarean delivery. Furthermore, postpartum complications were noted, including neonatal intensive care unit (NICU) admission, a low appearance, pulse, grimace, activity, and respiration (APGAR) score, macrosomia, postdate, and post-term delivery were higher among these pregnant women [24-25]. This study focused only on three complications: CS, preterm, and post-term deliveries. 

The present study showed an increased frequency of CS among obese pregnant women with a total of 1,150 cases. This finding was higher than any previous studies conducted locally [7, 21]. This might be secondary to different sampling pools in the studies. Nevertheless, the pregnant subjects were also found to be at higher risk of preterm delivery. In this study, there was a total of 305 preterm neonates. Studies conducted in the Eastern region of SA had a lower prevalence than our study, with 28 cases combined [7, 21]. Furthermore, this study had a higher frequency of post-term delivery, with a total of 48 cases compared to the previously mentioned study in the Eastern region with 11 cases.

As is the case with retrospective studies, these findings are not possible for generalization as it only represents pregnant women who were followed in the Obstetric Clinics at KAUH in the Western region of Saudi Arabia. Moreover, this study assessed a lesser number of pregnancy outcomes (mode of delivery, preterm, and post-term deliveries), and most of our findings were seen in obesity Class I, which can misrepresent the whole sample. However, the observation of these findings and complications remains to be validated by previous studies. 

## Conclusions

As it was shown, this study demonstrated that obesity in pregnant women coincides with an increased rate of CS, preterm, and post-term deliveries. Also, this study affirms that obesity is an obstacle that needs to be overcome with regular and customized care according to each patient's needs, especially for high-risk groups. Health education should include close monitoring of their weight before and during pregnancy. Weight reduction through diet and exercise is recommended with a dietitian. A nationwide study is required for a better assessment for each weight group and a recommended approach.
